# Differing severities of acute exacerbations of idiopathic pulmonary fibrosis (IPF): insights from the INPULSIS® trials

**DOI:** 10.1186/s12931-019-1037-7

**Published:** 2019-04-11

**Authors:** Michael Kreuter, Harald Koegler, Matthias Trampisch, Silke Geier, Luca Richeldi

**Affiliations:** 1 Center for Interstitial and Rare Lung Diseases, Pneumology and Respiratory Care Medicine, Thoraxklinik, University of Heidelberg, Röntgenstraße 1, 69120 Heidelberg, Germany; 2 Boehringer Ingelheim International GmbH, Ingelheim am Rhein, Germany; 3 Università Cattolica del Sacro Cuore, Fondazione Policlinico A. Gemelli, Rome, Italy

**Keywords:** Nintedanib, Tyrosine kinase inhibitor, Serious adverse events, Disease progression, Treatment outcome

## Abstract

**Background:**

Given the broad definition of an acute exacerbation of IPF, it is likely that acute exacerbations are heterogeneous in their aetiology, severity and clinical course. We used pooled data from the INPULSIS® trials of nintedanib versus placebo to investigate whether acute exacerbations reported as serious adverse events were associated with higher mortality than those reported as non-serious adverse events and to assess the effect of nintedanib on these types of events.

**Methods:**

Adverse events considered by an investigator to be an acute exacerbation were adjudicated as a confirmed acute exacerbation, suspected acute exacerbation, or not an acute exacerbation. Time to first investigator-reported acute exacerbation or confirmed/suspected acute exacerbation reported as a serious adverse event or non-serious adverse event over the 52-week treatment period was assessed *post-hoc*. Deaths were assessed based on data collected over the 52-week treatment period.

**Results:**

Of 63 patients who had ≥1 investigator-reported acute exacerbation, 48 (76.2%) had a first acute exacerbation reported as a serious adverse event. Thirty-six (3.4%) patients had ≥1 confirmed/suspected acute exacerbation, of whom 31 had a first event reported as a serious adverse event. Investigator-reported acute exacerbations reported as serious adverse events occurred in 23 patients in the nintedanib group and 26 in the placebo group. Confirmed/suspected acute exacerbations reported as serious adverse events occurred in 10 and 21 patients in these groups, respectively. Nintedanib significantly reduced the risk of a first acute exacerbation reported as a serious adverse event (HR 0.57 [95% CI: 0.32, 0.99]; *p* = 0.0476) and the risk of a first confirmed/suspected acute exacerbation reported as a serious adverse event (HR 0.30 [95% CI: 0.14, 0.64]; *p* = 0.0019) versus placebo. A higher proportion of patients with investigator-reported acute exacerbations reported as serious adverse events died than patients with acute exacerbations reported as non-serious adverse events (61.2% versus 7.1%).

**Conclusion:**

Different severities of acute exacerbation of IPF may exist. Acute exacerbations reported as serious adverse events in the INPULSIS® trials were associated with high mortality. Nintedanib significantly reduced the risk of acute exacerbations reported as serious adverse events.

**Trial registration:**

ClinicalTrials.gov NCT01335464 and NCT01335477.

## Introduction

Idiopathic pulmonary fibrosis (IPF) is a chronic progressive fibrosing interstitial lung disease characterised by loss of lung function [1]. IPF has a variable and unpredictable clinical course [2]. Many patients experience acute deteriorations in lung function known as acute exacerbations, which are associated with very high morbidity and mortality [3]. A perspective published by an international group of experts in 2007 defined acute exacerbations of IPF as acute deteriorations in respiratory symptoms associated with changes in radiographical appearance and the absence of an identifiable cause [4]. In 2016, an international working group proposed that the requirement for an event to be idiopathic be removed from the definition of an acute exacerbation of IPF based on recognition that there is little clinical or biological rationale for distinguishing idiopathic from non-idiopathic events [3].

The reported frequency of acute exacerbations in patients with IPF varies widely depending on the methodology used. In clinical trials conducted in patients with IPF and mild to moderate impairment in lung function at baseline, acute exacerbations were reported in 2 to 16% of placebo-treated patients over 24 to 60 weeks [5–17]. Similar or slightly higher frequencies of acute exacerbations have been observed in contemporary cohort studies [18–21].

Given the broad definition of an acute exacerbation of IPF, it is likely that events classified as acute exacerbations of IPF are heterogeneous in their aetiology, severity, and clinical course. A clinical trial, in which adverse event reporting rules provide a definition for a serious adverse event, represents an opportunity to assess the clinical impact of acute exacerbations based on their seriousness. In this analysis, we used data from the Phase III INPULSIS® trials of nintedanib in patients with IPF to investigate whether acute exacerbations reported as serious adverse events were associated with higher mortality than events reported as non-serious adverse events and to assess the effect of nintedanib on these events.

## Methods

The design of the two replicate INPULSIS® trials has been described [17]. Briefly, patients aged ≥40 years with a diagnosis of IPF within the previous 5 years, forced vital capacity (FVC) ≥50% predicted, diffusing capacity of the lung for carbon monoxide (DLco) 30–79% predicted and a forced expiratory volume in 1 s/FVC ratio ≥ 0.7 were randomised 3:2 to receive nintedanib 150 mg twice daily or placebo for 52 weeks, with a follow-up visit 4 weeks after the last dose of trial medication. The primary endpoint was the annual rate of decline in FVC (mL/year). Time to first investigator-reported acute exacerbation over the 52-week treatment period was a key secondary endpoint. Acute exacerbations were defined in the trial protocol as events meeting all of the following criteria: worsening/development of dyspnoea within 30 days; new diffuse pulmonary infiltrates on chest X-ray and/or high-resolution computed tomography parenchymal abnormalities with no pneumothorax or pleural effusion (new ground-glass opacities) since last visit; and exclusion of known causes of acute worsening, including infection, left heart failure, and pulmonary embolism, as per routine clinical practice and microbiological studies. Adverse events considered by an investigator to be acute exacerbations were adjudicated by a committee of three experts blinded to treatment assignment as a confirmed acute exacerbation (if all protocol-defined criteria were met), a suspected acute exacerbation (if the event was felt to be an acute exacerbation but did not meet all protocol-specified criteria), or not an acute exacerbation (if an alternative cause was identified) [22].

Adverse events were reported by the investigators from randomisation to 4 weeks after the last dose of trial medication. Serious adverse events were defined as events that resulted in death, were immediately life-threatening, resulted in persistent or clinically significant disability/incapacity, required/prolonged hospitalisation, were related to a congenital anomaly or birth defect, or were deemed serious for any other reason.

We conducted a *post-hoc* analysis of time to first acute exacerbation reported as a serious adverse event or non-serious adverse event over the 52-week treatment period using pooled data from both INPULSIS® trials. Hazard ratios (HR) and two-sided *p*-values were calculated using Cox’s regression models with terms for trial, treatment, sex, age, height. Baseline characteristics of patients with ≥1 investigator-reported acute exacerbation reported as a serious adverse event versus patients who did not have an acute exacerbation were compared using t-test (continuous variables) or chi-square test (categorical variables). Deaths were based on data collected over the 52-week treatment period.

## Results

A total of 1061 patients were treated in the INPULSIS® trials (638 with nintedanib, 423 with placebo). At baseline, mean FVC was 80% predicted and mean DLco was 47% predicted. The majority of patients were male (79%) and former smokers (68%).

Over 52 weeks, 63 (5.9%) patients had ≥1 investigator-reported acute exacerbation, of whom 48 (76.2%) had a first acute exacerbation reported as a serious adverse event (Table 1). Of these 48 events, 20 had fatality noted as the reason for the event being regarded as a serious adverse event. One patient had an acute exacerbation reported as a non-serious adverse event followed by an acute exacerbation reported as a serious adverse event; in subsequent analyses, this patient was classified according to his worse event. Thirty-six (3.4%) patients had ≥1 adjudicated confirmed/suspected acute exacerbation, of whom 31 had a first event reported as a serious adverse event. Twenty-seven (2.5%) patients had an acute respiratory worsening adjudicated as not an acute exacerbation, of whom 18 had a first event reported as a serious adverse event.

**Table 1:** Acute exacerbations in the INPULSIS® trials

	Adjudication result		
	Confirmed/suspected acute exacerbation	Not an acute exacerbation	Total
Adverse event category			
Serious adverse event	31	18	49*
Non-serious adverse event	5	9	14*
Total	36	27	63

*One patient had an acute exacerbation reported as a non-serious adverse event followed by an acute exacerbation reported as a serious adverse event. In subsequent analyses, this patient was classified according to his worse event

Patients with ≥1 investigator-reported acute exacerbation reported as a serious adverse event had significantly lower FVC and DLco and higher (worse) St George’s Respiratory Questionnaire (SGRQ) total score, composite physiological index and Gender-Age-Physiology (GAP) stage at baseline than patients who did not have an investigator-reported acute exacerbation (Table 2).

**Table 2:** Baseline characteristics by acute exacerbation reported in INPULSIS® trials (mean ± SD or n %)

	Patients who did not have an acute exacerbation (*n* = 998)	Patients who had ≥1 acute exacerbation reported as a serious adverse event (*n* = 49)	Patients who had ≥1 acute exacerbation but none reported as a serious adverse event (*n* = 14)	*p* value for patients who did not have an acute exacerbation vs patients who had ≥1 acute exacerbation reported as a serious adverse event
Male	789 (79.1)	43 (87.8)	9 (64.3)	n.s.
Age, years	66.6 ± 8.0	69.1 ± 7.8	69.8 ± 6.7	n.s.
Body mass index, kg/m^2^	28.0 ± 4.6	27.0 ± 4.1	27.2 ± 4.2	n.s.
Ex/current smoker	717 (71.8)	39 (79.6)	9 (64.3)	n.s.
Centrilobular emphysema*	401 (40.2)	17 (34.7)	2 (14.3)	n.s.
Time since diagnosis, years	1.6 ± 1.3	1.8 ± 1.5	1.9 ± 1.3	n.s.
FVC, mL	2746 ± 780	2372 ± 628	2057 ± 549	0.001
FVC, % predicted	80.2 ± 17.8	71.5 ± 15.9	62.0 ± 7.9	0.0008
Diffusing capacity of the lung for carbon monoxide^†^, mmol/min/kPa	3.9 ± 1.2	3.4 ± 1.3	3.3 ± 1.0	0.0074
St. George’s Respiratory Questionnaire total score	38.9 ± 18.8	50.6 ± 17.9	45.3 ± 18.2	< 0.0001
Composite physiological index^‡^	45.7 ± 10.8	51.3 ± 11.8	54.9 ± 8.3	0.0004
GAP score^§^	3.5 ± 1.3	4.2 ± 1.3	4.6 ± 1.0	0.0002
HRCT assessment^‖^				
Criteria A, B and C met	432 (43.3)	25 (51.0)	6 (42.9)	n.s.
Criteria A and C met	99 (9.9)	5 (10.2)	0 (0.0)	n.s.
Criteria B and C met	442 (44.3)	19 (38.8)	7 (50.0)	n.s.

*Based on qualitative assessment of HRCT scans, centrally reviewed by a single radiologist. ^†^Haemoglobin-corrected. ^‡^As described in [28]. ^§^As described in [29]. ^‖^A: definite honeycomb lung destruction with basal and peripheral predominance; B: presence of reticular abnormality and traction bronchiectasis consistent with fibrosis with basal and peripheral predominance; C: absence of atypical features, specifically nodules and consolidation, with ground glass opacity, if present, less extensive than reticular opacity pattern. n.s., not significant

**Fig. 1:**
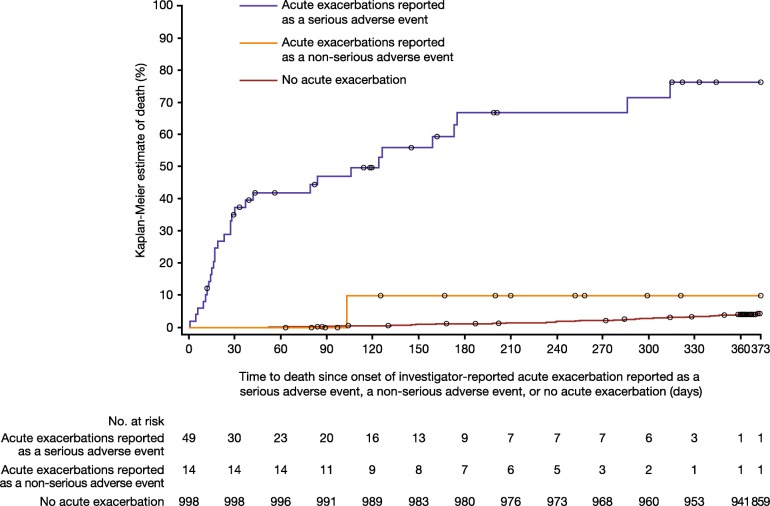
Time to death since onset of first investigator-reported acute exacerbation reported as a serious adverse event, first acute exacerbation reported as a non-serious adverse event, and in patients with no acute exacerbation. Thirteen patients with no acute exacerbation died beyond day 372

Deaths occurred in a higher proportion of patients with investigator-reported acute exacerbations reported as serious adverse events (*n* = 30, 61.2%) than patients with acute exacerbations reported as non-serious adverse events (*n* = 1; 7.1%) or patients who did not have an acute exacerbation (*n* = 43; 4.3%). There was a significant difference in the risk of death between patients with acute exacerbations reported as serious adverse events and patients who did not have an acute exacerbation (HR 53.6 [95% CI: 32.1, 89.5]; *p* < 0.001) and a numerical, but not statistically significant, difference in the risk of death between patients with acute exacerbations reported as non-serious adverse events and patients who did not have an acute exacerbation (HR 3.7 [95% CI: 0.5, 27.2]; *p* = 0.1962) (Fig. 1).

Investigator-reported acute exacerbations reported as serious adverse events occurred in 23 (3.6%) versus 26 (6.1%) patients in the nintedanib and placebo groups, respectively. Of these patients, 13 (56.5%) in the nintedanib group and 17 (65.4%) in the placebo group died. Investigator-reported acute exacerbations reported as non-serious adverse events occurred in 8 (1.3%) versus 6 (1.4%) patients in the nintedanib and placebo groups, respectively. Of these, no patients in the nintedanib group and 1 patient in the placebo group died.

Confirmed/suspected acute exacerbations reported as serious adverse events occurred in 10 (1.6%) versus 21 (5.0%) patients in the nintedanib and placebo groups, respectively. Of these patients, 5 (50.0%) in the nintedanib group and 14 (66.7%) in the placebo group died. Confirmed/suspected acute exacerbations reported as non-serious adverse events occurred in 2 (0.3%) versus 3 (0.7%) patients in the nintedanib and placebo groups, respectively. Of these, no patients in the nintedanib group and 1 patient in the placebo group died.

**Fig. 2:**
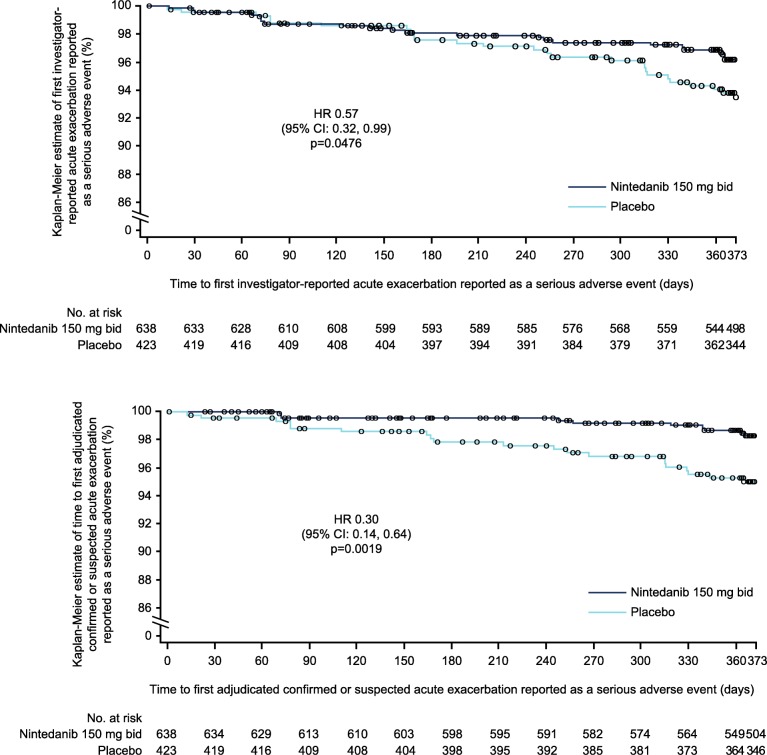
Time to (**a**) first investigator-reported and (**b**) adjudicated confirmed or suspected acute exacerbation reported as a serious adverse event in patients treated with nintedanib or placebo

Compared to placebo, nintedanib significantly reduced the risk of a first acute exacerbation reported as a serious adverse event (HR 0.57 [95% CI: 0.32, 0.99]; *p* = 0.0476) and the risk of a first confirmed/suspected acute exacerbation reported as a serious adverse event (HR 0.30 [95% CI: 0.14, 0.64]; *p* = 0.0019) (Fig. 2).

## Discussion

Acute exacerbations in patients with IPF are known to be associated with high mortality [3]. A retrospective review of clinical records from 553 patients with IPF in Japan from 2003 to 2007 found that acute exacerbation was the cause of death in 40% of patients [23]. Previous analyses of data from the INPULSIS® trials have shown that the mortality rate following an investigator-reported acute exacerbation in placebo-treated patients was 40% after 30 days and 57% after 180 days and that mortality following investigator-reported acute exacerbations and adjudicated confirmed/suspected acute exacerbations was similar [22]. In these new analyses, we demonstrated that acute exacerbations reported as serious adverse events are, regardless of adjudication, associated with particularly high mortality, with 61% of patients who had such an event dying within the 52-week treatment period. This suggests that while all acute exacerbations are clinically significant events, some types of acute exacerbations, possibly those with specific aetiologies or those that affect patients with certain clinical characteristics, might have a particularly adverse impact on prognosis.

Patients who had acute exacerbations reported as serious adverse events had more severe functional impairment at baseline than patients who did not have an acute exacerbation. This is in line with previous analyses of risk factors for acute exacerbations, which have consistently found a higher rate of acute exacerbations in patients with lower FVC at baseline [21, 22, 24, 25], and supports the concept of acute exacerbations as clinical events triggered by injury or stress to the lung that are more likely to manifest in patients with advanced disease [3].

Nintedanib is an intracellular inhibitor of tyrosine kinases that has been shown to inhibit processes fundamental to the pathogenesis of IPF [26]. In clinical trials, nintedanib reduced disease progression in patients with IPF by reducing the rate of decline in FVC by about 50%, irrespective of FVC at baseline [16, 17, 27]. Pooled data from the INPULSIS® trials showed that investigator-reported acute exacerbations were reported in 4.9 and 7.6% of patients treated with nintedanib and placebo, respectively (HR 0.64 [95% CI: 0.39, 1.05]; *p* = 0.08) and confirmed/suspected acute exacerbations in 1.9 and 5.7% of patients treated with nintedanib and placebo, respectively (HR 0.32 [95% CI: 0.16, 0.65]; *p* = 0.001) [17]. A further *post-hoc* analysis suggested that mortality following investigator-reported acute exacerbations and confirmed/suspected acute exacerbations was lower in patients treated with nintedanib than placebo (HR 0.56 [95% CI: 0.28, 1.11]; *p* = 0.092 and HR 0.57 [95% CI: 0.22, 1.48; *p* = 0.24, respectively) [22].

Limitations of our analyses include their *post-hoc* nature, the small number of events, and the fact that events that resulted in death should, by definition, have been reported as serious adverse events. We were unable to determine the reasons why some events that were reported as acute exacerbations were not reported as serious adverse events, particularly when all events that resulted in hospitalization were to be reported as serious adverse events. Although acute exacerbations that were reported as serious adverse events were associated with particularly high mortality, it is unknown whether these events were also associated with other indicators of severity such as treatment in intensive care.

## Conclusions

Our analysis of data from the Phase III INPULSIS® trials of nintedanib in patients with IPF showed that acute exacerbations reported as serious adverse events were associated with higher mortality than events reported as non-serious adverse events. Nintedanib significantly reduced the risk of acute exacerbations reported as serious adverse events. Further research is needed into the causes of acute exacerbations in patients with IPF and how their risk and impact can be reduced. A severity grading might aid clinicians in making decisions about how to manage acute exacerbations in patients with IPF.

## Abbreviations

DLco: Diffusing capacity of the lung for carbon monoxide
FVC: Forced vital capacity
GAP: Gender-Age-Physiology
HR: Hazard ratio
IPF: Idiopathic pulmonary fibrosis
SGRQ: St George’s Respiratory Questionnaire
